# Application of the dyadic data analysis in behavioral medicine research: marital satisfaction and anxiety in infertile couples

**DOI:** 10.1186/s12874-018-0582-y

**Published:** 2018-10-26

**Authors:** Saman Maroufizadeh, Mostafa Hosseini, Abbas Rahimi Foroushani, Reza Omani-Samani, Payam Amini

**Affiliations:** 1 Department of Epidemiology and Reproductive Health, Reproductive Epidemiology Research Center, Royan Institute for Reproductive Biomedicine, ACECR, Tehran, Iran; 2 Department of Epidemiology and Biostatistics, School of Public Health, Tehran University of Medical Sciences, Tehran, Iran

**Keywords:** Dyadic data analysis, Dyad, Anxiety, Marital satisfaction, Infertility

## Abstract

**Background:**

Dyadic data analysis (DDA) is increasingly being used to better understand, analyze and model intra- and inter-personal mechanisms of health in various types of dyads such as husband-wife, caregiver-patient, doctor-patient, and parent-child. A key strength of the DDA is its flexibility to take the nonindependence available in the dyads into account. In this article, we illustrate the value of using DDA to examine how anxiety is associated with marital satisfaction in infertile couples.

**Methods:**

This cross-sectional study included 141 infertile couples from a referral infertility clinic in Tehran, Iran between February and May 2017. Anxiety and marital satisfaction were measured by the anxiety subscale of the Hospital Anxiety and Depression Scale and 10-Item ENRICH Marital Satisfaction Scale, respectively. We apply and compare tree different dyadic models to explore the effect of anxiety on marital satisfaction, including the Actor-Partner Interdependence Model (APIM), Mutual Influence Model (MIM), and Common Fate Model (CFM).

**Results:**

This study demonstrated a practical application of the dyadic models. These dyadic models provide results that appear to give different interpretations of the data. The APIM analysis revealed that both men’s and women’s anxiety excreted an actor effect on their own marital satisfaction. In addition, women’s anxiety exerted a significant partner effect on their husbands’ marital satisfaction. In MIM analysis, in addition to significant actor effects of anxiety on marital satisfaction, women’s reports of marital satisfaction significantly predicted men’s marital satisfaction. The CFM analysis revealed that higher couple anxiety scores predicted lower couple marital satisfaction scores.

**Conclusion:**

In sum, the study highlights the usefulness of DDA to explore and test the phenomena with inherently dyadic nature. With regard to our empirical data, the findings confirmed that marital satisfaction was influenced by anxiety in infertile couples at both individual and dyadic level; thus, interventions to improve marital satisfaction should include both men and women. In addition, future studies should consider using DDA when dyadic data are available.

## Background

Infertility is an ongoing public health problem and is estimated to affect 9% of reproductive-aged couples worldwide [1]. Besides being a medical condition in itself, infertility is a social condition that can lead to considerable social and psychological distress [2, 3]. Among psychological distress, anxiety is one of the most frequent psychiatric disorders in infertile patients. For example, in two studies conducted in Iran, the prevalence of anxiety and genialized anxiety disorder were 49.6% and 33.0%, respectively, which are considerably higher than what was reported in general population [4, 5]. One the other hand, it has been frequently reported that infertility and its treatments can cause a negative effect on one’s marital satisfaction [6, 7]. Also, some previous studies have found that subclinicals anxiety symptoms was negatively associated with marital satisfaction [8]. Since infertility is a shared condition within the couple [9], considering both members of the couple in the analysis of their relationships is necessary.

On the other hand, one of the basic assumptions underlying statistical analysis is the independence of observations. However, the dyadic designs (e.g., research on man-woman dyads and caregiver-patient dyads) create observations that are not independent which make the conventional statistical methods as inappropriate analysis tools [10]. This type of design is usually applicable when researchers want to understand relationship processes between two people. Some of these processes are inherently dyadic processes, and one of the special characteristics of these processes is dyadic nonindependence. Conceptually, dyadic nonindependence is defined as “if the two scores from the two members of the dyad are non-independent, then those two scores are more similar to (or different from) one another than are two scores from two people who are not members of the same dyad” [10]. Dyadic nonindependence occurs in a variety of contexts that involve two people, such as relationship processes studies in husband-wife, doctor-patient, caregiver-patient, parent-child, siblings and friends. In these conditions, statistical analytical techniques that take non-independence into account is required. To address this issue, Kenny et al. [10] developed three important classes of dyadic models: the Actor-Partner Interdependence Model (APIM), Mutual Influence Model (MIM), and Common Fate Model (CFM). A detailed description of these models is presented in the Methods section.

Most of the studies with inherently dyadic nature in the infertility context and other behavioral medicine have been analyzed using individual as the unit of analysis rather than dyads. Although valuable, these studies fail to consider the dyadic non-independency of the data resulting in biased estimates of associations [11]. However, in the last two decades, a great number of researchers have started to develop dyadic data analysis (DDA) techniques that consider dyadic nonindependence as a source of information rather than attempt to control for it [12, 13]. In the context of infertility, APIM framework is by far the most widely used model to examine many relationship processes, such as effect of severe depressive symptoms on infertility-related distress [14], attachment patterns on perceived infertility stress [15], and the role of attachment anxiety and attachment avoidance on the psychosocial well-being [16]. Although, the DDA are being used more and more in a variety of researches as a framework for investigating dyadic processes [17–19], many of researchers still require more information on how to analyze the data in a way that optimizes its value [13]. Thus, the aim of this article is twofold. First, we explain how DDA can aid our understanding of dyadic nature of phenomena in medical research. Second, we used three different dyadic models (i.e. APIM, MIM, and CFM) to examine how anxiety is associated with marital satisfaction in infertile couples. In this study, we examine the following research hypothesis:

*APIM analysis:* (1) One’s level of anxiety is associated with his/her own level of marital satisfaction (actor effects); (2) One’s level of anxiety is associated with his/her spouse’s level of marital satisfaction (partner effects); (3) There is a significant difference between male and female actor effects of anxiety on marital satisfaction; (4) There is a significant difference between male and female partner effects of anxiety on marital satisfaction. *MIM analysis:* (5) There is a dyadic feedback effect between males and females’ marital satisfaction; (6) There is a significant difference between male and female dyadic feedback effects of marital satisfaction. *CFM analysis:* (7) Couple’s anxiety is associated with couple’s marital satisfaction.

## Methods

### Participants and study design

This was a cross-sectional study of a sample of infertile couples seeking infertility treatment from Tehran, Iran between February and May 2017. Couples who met the following criteria were invited to participate in this study: experiencing infertility problems; age over 18 years; ability to read and write in Persian. Infertility is defined as “the failure to achieve a clinical pregnancy after 12 months or more of regular unprotected sexual intercourse” [20]. Couples were instructed to fill out the instruments separately from each other in a quiet place. It took about 5 min to complete the questionnaires. In total, 141 infertile couples agreed to participate and filled out the instruments completely (response rate: 82.3%).

### Measures

#### Marital satisfaction

Marital satisfaction was measured by the 10-Item ENRICH Marital Satisfaction Scale (EMS Scale) [21]. Respondents rate items on a 5-point Likert-type scale ranging from 1 (strongly disagree) to 5 (strongly agree). After recoding the negative items, scores are summed to yield a total EMS Scale score ranging from 10 to 50, with higher scores indicating greater levels of marital satisfaction. For this study, the Cronbach’s alpha coefficient of the EMS Scale for males and females were 0.752 and 0.790, respectively.

#### Anxiety

Anxiety was measured by the anxiety subscale of the Hospital Anxiety and Depression Scale (HADS). The HADS is a widely used self-report instrument consisting 14 items designed to measure both anxiety (HADS-A) and depression (HADS-D) [22]. Items are rated on a 4-point Likert scale, ranging from 0 (no symptoms) to 3 (severe symptoms). Both subscales scores range from 0 to 21, with higher scores indicating greater levels of anxiety and depression. The Persian version of HADS has been validated [23] and widely used among infertile patients [4, 23]. For this study, the Cronbach’s alpha coefficients of the HADS-A for males and females were 0.841 and 0.865, respectively.

### Statistical analysis

As mentioned above, there are three main dyadic models for the analysis of the dyadic design. In this section, we describe these models in terms of the simplest scenario, one in which there is one independent variable (X) and one dependent variable (Y) for each member of a dyad, or four variables altogether.

#### APIM framework

**Fig. 1:**
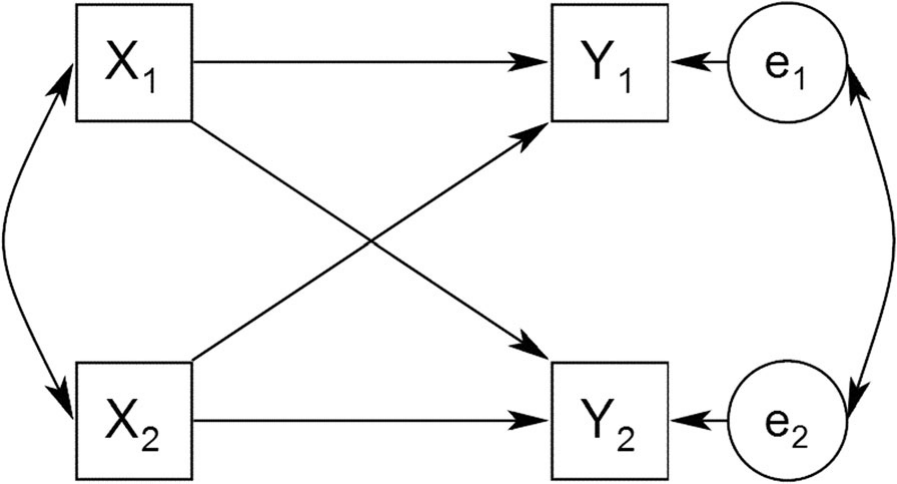
The Actor-Partner Interdependence Model (APIM). Note. X_1_ and X_2_ are predictor variables for person 1 and person 2, Y_1_ and Y_2_ are their respective outcome variables, and e_1_ and e_2_ are the corresponding error terms. The effect of a person’s X on his or her own Y is an actor effect. The effect of a person’s X on the partner’s Y is a partner effect [11]

The APIM is the most widely used model in dyadic research which developed by Kenny [10, 13]. This model is useful in determining intrapersonal effect (actor effect) and interpersonal effect (partner effect). Figure 1 depicts the basic APIM for Member 1 and Member 2. The path from the person’s X to that person’s Y is called actor effect and the path to the other person’s Y is called partner effect. In other words, the actor effect measures the degree to which a person’s outcome is influenced by his/her own characteristics, whereas the partner effect measures the degree to which a person’s outcome is influenced by his or her partner’s characteristics. As an example, consider the effects of anxiety on marital satisfaction in men-women dyads with infertility problem. It may be that one’s anxiety influences both her/his own and spouse’s marital satisfaction. This model has two actor effects a_1_ and a_2_ (horizontal arrows), and two partner effects p_12_ and p_21_ (diagonal arrows). In addition, there are also two major correlations in the APIM. The two X variables might be correlated, indicating probably compositional effect. The second correlation is the residual nonindependence in the Y variables, which represents the nonindependence not explained by the APIM.

Multilevel modeling (MLM) and structural equation modeling (SEM) are the dominant methods to estimate the APIM. As recommended in the literature [10, 24], SEM approach is the simplest method for estimating the APIM in case of distinguishable dyads. Furthermore, in this study, to examine whether the actor effects as well as the partner effect differed between men and women, we constrained these two coefficients to be equal and compared the chi-square statistics for the constrained and unconstrained (saturated) models.

#### MIM framework

**Fig. 2:**
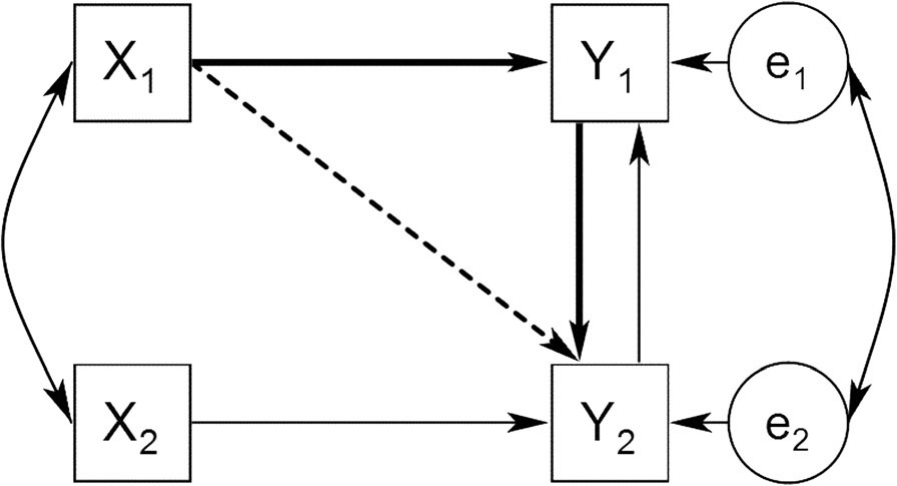
The Mutual Influence Model (MIM). Note. X_1_ and X_2_ are predictor variables for person 1 and person 2, Y_1_ and Y_2_ are their respective outcome variables, and e_1_ and e_2_ are the corresponding error terms. Each person’s outcome (Y) influences the other’s outcome. The effect of each actor’s independent variable on the partner’s outcome (Y) is mediated by the actor’s own outcome (see bolded arrows). The dashed line represents the indirect effect of the actor on the partner [11]

Like the APIM framework, the MIM is a model of interdependence because it models interpersonal effects (see Fig. 2). However, in the MIM framework, both persons’ outcomes directly influence one another (i.e. there is a dyadic feedback). Contrary to the APIM, the other cause of each person’s Y variable is not the partner’s X variable, but the partner’s Y variable. As one example, the MIM framework is useful to investigate marital satisfaction because of the reciprocity of this concept in relationship researches [25, 26]. The details of the method of estimation for MIM are discussed in Ref [10, 27].

#### CFM framework

**Fig. 3:**
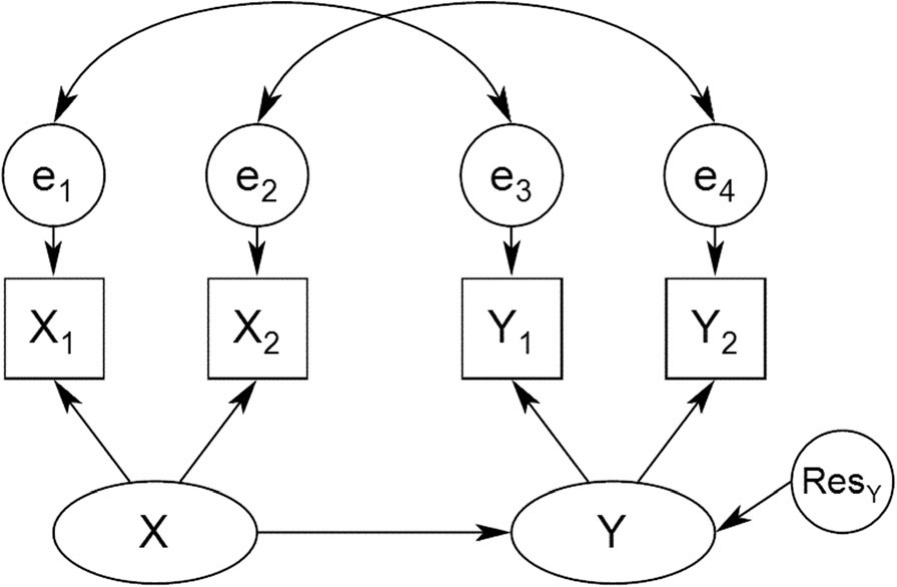
The Common Fate Model (CFM). Note. X_1_, X_2_, Y_1_, and Y_2_ denote manifest variables measured in person 1 and person 2; X and Y indicate latent variables. The latent predictor variable (X) influences the latent outcome variable (Y). Residuals (e.g., e_1_ and e_3_) are correlated within person. Res_Y_ is the residual on the latent outcome variable [11]

The third source that may produce nonindependence in dyads is common fate. Common fate occurs when both dyad members are affected by the same factor, such as environmental, cultural, or structural elements, or shared experiences [28]. For example, a parent and a child may be similar in some respect because they share a certain gene [29]. In the CFM, the covariation between dyad members’ scores is assumed to be due to some unmeasured factor that impacts both dyad members. In other words, the dyad members do not impact each other, but, rather, the same external force impacts both. As depicted in Fig. 3, in the CFM, the causal effect from X to Y occurs between latent variables. These latent variables are assumed to be variables that affect both members of the dyad. The details of the CFM and discussion of variables useful for this model are presented in in Ref. [28, 30–32].

#### Statistical software and model fit

We conducted structural equation modeling (SEM) using maximum likelihood estimation to test the models. Overall model fit was evaluated using multiple fit indices, as suggested in the literature. Specifically, five goodness-of-fit indices were used, including chi-square/degree of freedom (χ^2^/df), Tucker-Lewis Index (TLI), comparative fit index (CFI), root mean square error of approximation (RMSEA), and standardized root mean square residual (SRMR). Values of χ^2^/df < 2, TLI and CFI > 0.95, and RMSEA and SRMR< 0.08 are indicative of a good fit with the data [33–36].

All preliminary analyses were done with IBM SPSS Statistics for Windows, Version 22.0 (IBM Crop., Armonk, NY, USA) and DDA was done with Mplus software version 6.12 (Muthén & Muthén, Los Angeles, CA, USA).

## Results

### Characteristics of the infertile couples

Demographic and fertility characteristics of the infertile couples are outlined in Table 1. The husband, on average, were 5.10 years older than their wives (t _(140)_ = 12.88, *P* < 0.001), but they had a similar level of education as their wives (χ^2^_(1)_ = 2.56, *P* = 0.109). The average of infertility duration was 4.85 ± 3.76 years. The causes of infertility were as follows: male factor (36.2%), female factor (21.3%), both (19.1%), and unexplained (23.4%). The majority of the couples had primary infertility (72.3%), and no history of abortion (76.6%) and half of them experience at least one failure in previous infertility treatments.

**Table 1:** Demographic and fertility characteristics of the infertile couples (*n* = 141 couples)

	Men	Women	Test statistic	P
Age (years)	34.92 ± 6.35	29.82 ± 6.00	t_(140)_ = 12.88	< 0.001
Educational level			χ^2^_(1)_ = 2.56	0.109
Non-academic	96 (68.1)	85 (60.3)		
Academic	45 (31.9)	56 (39.7)		
Duration of marriage (years)	7.37 ± 4.40	–		
Duration of infertility (years)	4.85 ± 3.76	–		
Cause of infertility				
Male factor	51 (36.2)	–		
Female factor	30 (21.3)	–		
Both	27 (19.1)	–		
Unexplained	33 (23.4)	–		
History of ART treatment failure				
No (First treatment)	71 (50.4)	–		
Yes	70 (49.6)	–		
History of abortion				
No	108 (76.6)	–		
Yes	33 (23.4)	–		
Type of infertility				
Primary	102 (72.3)	–		
Secondary	39 (27.7)	–		

Values are given as mean ± SD or n (%)

*ART* Assisted Reproductive Technology

### Preliminary analyses

Means, SDs, and correlations for study variables are given in Table 2. There was no significant difference between men’s and women’s marital satisfaction (t _(140)_ = 0.09, *P* = 0.925). Women, however, reported higher anxiety than men (t _(140)_ = 3.39, *P* < 0.001). As seen in Table 2, men’s anxiety was correlated with their own as well as their wives’ marital satisfaction. Women’s anxiety was also correlated with their own as well as their husbands’ marital satisfaction. In addition, the within-dyad correlations between marital satisfaction and anxiety were direct and significant, indicating that if one partner was satisfied with their martial satisfaction and had high level of anxiety, the other one was also.

**Table 2:** Means, standard deviations, and correlations among study variables (*n* = 141 couples)

	Mean (SD)	1	2	3	4
1 Men marital satisfaction	39.31 (6.56)	1			
2 Men anxiety	6.52 (4.30)	−0.336^***^	1		
3 Women marital satisfaction	39.26 (6.70)	0.423^***^	−0.159	1	
4 Women anxiety	8.09 (4.46)	−0.235^**^	0.209^*^	−0.316^***^	1

*SD* Standard Deviation

^*^P < 0.05; ^**^P < 0.01; ^***^P < 0.001

### Dyadic data analysis

#### APIM analyses

The results of the basic APIM (Model 1) and three variations of the model (Model 2, Model3, and Model 4) are presented in Table 3. With regard to actor effects in Model 1, men as well as women who were highly anxious were less satisfied with their marital relationship (b = − 0.457, *P* < 0.001; b = − 0.445, *P* < 0.001, respectively). In other words, after adjusting for partner effects, each 1-point increase in one’s own anxiety score was associated with a 0.46- and 0.45-point decrease in one’s own marital satisfaction among men and women, respectively. With regard to partner effects, men with highly anxious wives reported lower level of marital satisfaction (b = − 0.254, *P* = 0.031), whereas the partner effect of men’s anxiety on women’s marital satisfaction was not statistically significant (b = − 0.150, *P* = 0.235). In other words, after adjusting for the actor effect, a unit increase of the women’s anxiety was associated with a 0.25-point decrease in their husbands’ marital satisfaction. The basic APIM is fully saturated, thus goodness-of-fit indices were not interpreted.

**Table 3:** Results of the APIM framework relating anxiety to marital satisfaction among infertile couples

	Model 1		Model 2		Model 3		Model 4	
	Estimate	SE	Estimate	SE	Estimate	SE	Estimate	SE
Actor effects								
A_M_ → MS_M_	−.457^***^	.122	−.451^***a^	.083	−.485^***^	.112	−.451^***c^	.083
A_W_ → MS_W_	−.445^***^	.122	−.451^***a^	.083	−.418^***^	.113	−.451^***c^	.083
Partner effects								
A_W_ → MS_M_	−.254^*^	.117	−.257^*^	.108	−.206^*b^	.083	−.207^*d^	.083
A_M_ → MS_W_	−.150	.127	−.147	.116	−.206^*b^	.083	−.207^*d^	.083
Covariances								
A_M_ ↔ A_W_	3.988^*^	1.638	3.988^*^	1.638	3.988^*^	1.638	3.988^*^	1.638
Res MS_M_ ↔ Res MS_W_	13.997^***^	3.421	13.999^***^	3.422	14.040^***^	3.429	13.976^***^	3.425
Model fit								
χ^2^	–		.004		.334		.532	
df	–		1		1		2	
P	–		.947		.563		.766	
χ^2^/df	–		.004		.334		.266	
CFI	–		1.000		1.000		1.000	
TLI	–		1.000		1.000		1.000	
RMSEA	–		<.001		<.001		<.001	
SRMR	–		.002		.012		.023	

Res MS_M_ and Res MS_W_ are residual terms of MS_M_ and MS_W_, respectively

Note. *n* = 141. *SE* Standard Error, *CFI* Comparative Fit Index, *TLI* Tucker–Lewis Index, *RMSEA* Root Mean Square Error of Approximation, *SRMR* Standardized Root Mean Square Residual

*A*_*M*_ Men’s Anxiety, *A*_*W*_ Women’s Anxiety, *MS*_*M*_ Men’s Marital Satisfaction, *MS*_*W*_ Women’s Marital Satisfaction

^*^P < 0.05; ^**^P < 0.01; ^***^P < 0.001

^abcd^These coefficients were constrained to be equal

In order to further examine these effects, several equality constraint tests were conducted to compare actor and/or partner effects between men and women by the examination of the chi-square difference test. Constraining the actor effects to be equal (see Model 2 in Table 3) did not significantly worsen the model fit (χ^2^_(1)_ = 0.004, *P* = 0.947), showing that the actor effects of anxiety on marital satisfaction did not differ by gender. The other fit indices for this model were excellent (CFI = 1.000; TLI = 1.000; RMSEA< 0.001; and SRMR = 0.002). In Model 3, we compared the partner effects between men and women. Similar to Model 2, equal partner effects constraint (see Model 3 in Table 3) did not worsen the fit of the model compared to Model 1 (χ^2^_(1)_ = 0.334, *P* = 0.563). In addition, the two previous constraints from Models 2 and 3 were assumed in Model 4 which did not lead to a significance change in model fit (χ^2^_(2)_ = 0.532, *P* = 0.766).

#### MIM analyses

The results of the basic MIM (Model 5) and three variations of the model (Model 6, Model 7, and Model 8) are presented in Table 4. In the Model 5, men’s anxiety was significantly negatively related with their own marital satisfaction (b = − 0.371, *P* = 0.004). Likewise, women’s anxiety was significantly negatively related with their own martial satisfaction (b = − 0.361, *P* = 0.011). Further, women’s reports of marital satisfaction significantly predicted men’s marital satisfaction (b = 0.570, *P* = 0.024), whereas men’s marital satisfaction did not predict women’s marital satisfaction (b = 0.329, *P* = 0.202). The MIM also estimated the indirect effects of husbands’ and wives’ anxiety on their partners’ marital satisfaction. The indirect effect of wives’ anxiety on husbands’ marital satisfaction was negative and significant (b = − 0.206, *P* = 0.020), but indirect effect of husbands’ anxiety on wives’ marital satisfaction was not statistically significant (b = − 0.122, *P* = 0.140). Like the basic APIM (Model 5), the basic MIM is also fully saturated. In this framework, we performed several contrasts that were of theoretical importance. Like the Model 2, the first test constrained the actor effects for men and women to be equal (Model 6). This constraint did not lead to significant decrease in model fit (χ^2^_(1)_ = 0.004, *P* = 0.947). The second test constrained the dyadic feedback effects for men and women to be equal (Model 7). This constraint also did not lead to significantly poorer model fit (χ^2^_(1)_ = 0.514, *P* = 0.473). In addition, the two previous constraints from Models 6 and 7 were tested in Model 8 simultaneously. These constraints also did not lead to significant decrease in model fit (χ^2^_(2)_ = 0.532, *P* = 0.766).

**Table 4:** Results of the MIM framework relating anxiety to marital satisfaction among infertile couples

	Model 5		Model 6		Model 7		Model 8	
	Estimate	SE	Estimate	SE	Estimate	SE	Estimate	SE
Actor effects								
A_M_ → MS_M_	−.371^**^	.130	−.367^**a^	.115	−.368^*^	.144	−.357^**c^	.115
A_W_ → MS_W_	−.361^*^	.143	−.367^**a^	.115	−.349^**^	.127	−.357^**c^	.115
Dyadic feedback effects								
MS_W_ → MS_M_	.570^*^	.253	.570^*^	.249	.454^*b^	.190	.457^*d^	.188
MS_M_ → MS_W_	.329	.258	.326	.257	.454^*b^	.190	.457^*d^	.188
Covariances								
A_M_ ↔ A_W_	3.988^*^	1.638	3.988^*^	1.638	3.988^*^	1.638	3.988^*^	1.638
Res MS_M_ ↔ Res MS_W_	−18.067	12.583	−17.954	12.444	−17.812	12.555	−18.065	12.418
Model fit								
χ^2^	–		.004		.514		.532	
df	–		1		1		2	
P			.947		.473		.766	
χ^2^/df	–		.004		.514		.266	
CFI	–		1.000		1.000		1.000	
TLI	–		1.000		1.000		1.000	
RMSEA	–		<.001		<.001		<.001	
SRMR	–		.002		.020		.023	

Res MS_M_ and Res MS_W_ are residual terms of MS_M_ and MS_W_, respectively

Note. *n* = 141. *SE* Standard Error, *CFI* Comparative Fit Index, *TLI* Tucker–Lewis Index, *RMSEA* Root Mean Square Error of Approximation, *SRMR* Standardized Root Mean Square Residual

*A*_*M*_ Men’s Anxiety, *A*_*W*_ Women’s Anxiety, *MS*_*M*_ Men’s Marital Satisfaction, *MS*_*W*_ Women’s Marital Satisfaction

^*^P < 0.05; ^**^P < 0.01; ^***^P < 0.001

^abcd^These coefficients were constrained to be equal

#### CFM analysis

As mentioned above, the correlations between men and women were medium for marital satisfaction and relatively low for anxiety. Thus, these findings may be taken as supporting the assumption that infertile couples share common variance on these two constructs, particularly for marital satisfaction. In addition, the finding that actor and partner effects between anxiety and marital satisfaction were similar in magnitude and direction justifies the implementation of these dyadic variables in a CFM framework. To test the model in this framework (Model 9), we set all factor loadings of the two latent variables anxiety and marital satisfaction to 1. As expected, higher couple anxiety scores predicted lower levels of marital satisfaction (b = − 1.440, *P* = 0.012). This model fit the data well: χ^2^_(1)_ = 0.529, *P* = 0.467; CFI = 1.000; TLI = 1.000; RMSEA< 0.001; and SRMR = 0.017. The explained variance of marital satisfaction through anxiety was 44.1% (Table 5).

**Table 5:** Results of the CFM framework relating anxiety to marital satisfaction among infertile couples

	Model 9	
	Estimate	SE
Dyadic level effect		
A → MS	−1.440^*^	.571
Individual-level effects		
Res A_M_ ↔ Res MS_M_	−4.010^†^	2.321
Res A_W_ ↔ Res MS_W_	−3.388	2.382
Model fit		
χ^2^	.529	
df	1	
P	.467	
χ^2^/df	.529	
CFI	1.000	
TLI	1.000	
RMSEA	<.001	
SRMR	.017	

Res A_M_, Res A_W_, Res MS_M_, and Res MS_W_ are residual terms of A_M_, A_W_, MS_M_, and MS_W_, respectively

Note. *n* = 141. *SE* Standard Error, *CFI* Comparative Fit Index, *TLI* Tucker–Lewis Index, *RMSEA* Root Mean Square Error of Approximation, *SRMR* Standardized Root Mean Square Residual

*A*_*M*_ Men’s Anxiety, *A*_*W*_ Women’s Anxiety, *MS*_*M*_ Men’s Marital Satisfaction, *MS*_*W*_ Women’s Marital Satisfaction

^*^P < 0.05; ^†^*P* < 0.1

## Discussion

We used the APIM, MIM, and CFM frameworks to explore the associations among husbands’ and wives’ assessments of anxiety and marital satisfaction, using data gathered from 141 infertile couples who seek treatments for infertility in Tehran, Iran. The findings from these analyses all show that anxiety is related to marital satisfaction. The interpretation of that relationship, however, would be different depending on which analysis one performed.

Key findings from the APIM analyses were that (a) men’s and women’s report of their anxiety predicted changes in their marital satisfaction (actor effects). Therefore, the Hypothesis 1 was accepted; (b) men’s and women’s anxiety was associated with their wife’s and husband’s marital satisfaction, respectively (partner effect) (based on Model 3 and 4). Therefore, the Hypothesis 2 was accepted; (c) actor effects as well as partner effects of anxiety on marital satisfaction were similar between men and women. Therefore, the Hypotheses 3 and 4 were rejected. The key feature of this APIM analysis which cannot be examined by individual model analytical approach is the explore the interpersonal effects (i.e. partner effects).

Findings of the MIM framework indicated that husbands’ and wives’ marital satisfaction were considerably reciprocally correlated (thus the Hypothesis 5 was accepted), suggesting feedback, and the magnitude of this association was similar (thus the Hypothesis 6 was rejected). The MIM also can test whether husbands’ and wives’ anxiety are indirectly related with their partners’ marital satisfaction via their own marital satisfaction (i.e., mediation). Based on the MIM findings, the indirect effects of spouses’ anxiety on their partners’ marital satisfaction imply that marital satisfaction in infertile patients was influenced by not only their own anxiety, but also their spouses’ anxiety.

The CFM framework provided a valuable insight with regard to the couple-level process. The key finding from this framework was that anxiety symptoms was related with marital satisfaction at the dyadic level. In other words, higher couple anxiety scores predicted lower couple marital satisfaction. Therefore, the Hypothesis 7 was accepted. In the data, two of the key indicators for selecting CFM as a reasonable model were met. First, the interpartner (dyadic) correlations were greater than 0.2 and 0.4 for anxiety and marital satisfaction scores, respectively, which approximately satisfied the recommendations in the literature [28, 31] for variables that are thought to represent dyadic-level variation. Second, the result that actor and partner effects between anxiety and marital satisfaction were same in both direction and strength warrants the use of these variables in a CFM framework. Theoretical and methodological issues regarding the selecting of model are discussed in Ref [31, 37].

Regarding preliminary analysis, women’s anxiety was significantly higher than their husbands, indicating that women may be more considerably affected than men by infertility problem. This result is in line with previous research [38, 39]. Consistent with previous studies [40–42], marital satisfaction was unrelated to gender. This finding is also in line with two previous studies on infertile couples’ quality of life, measured by the Fertility Quality of Life (FertiQoL), which found that the FertiQoL-Relational scores did not differ across partners [43, 44]. However, in a study performed among infertile couples in Poland, women’s marital satisfaction was lower than their husbands [45].

Of all dyadic data analytic approaches, the APIM framework has been—and is likely to continue to be—favored by researchers. However, when measurements are taken on both members of a dyad, researchers must decide whether the process is best represented as interdependent or common fate. The APIM and MIM would be appropriate dyadic models that represent interdependent processes. In APIM framework, interdependence of the outcome variables is hypothesized to come about because of the predictor variables, whereas in MIM framework, interdependence of the outcome variables may come about because these outcome variables simultaneously affect each other [32]. The CFM is a dyadic model that is useful when both dyad members are affected by the common factor.

The current study offers a number of important contributions to the literature. Researches regarding the relationship between anxiety and marital satisfaction tend to focus on individuals, despite the obviously dyadic nature of marital satisfaction, particularly in the infertility context. Moreover, given the paucity of prior research on couples involved in committed marital relationships and shared health condition like infertility problem, the current research conducted among infertile married couples from a dyadic perspective. The results of this study have potentially important clinical implications. First, confirmation of the significance of the studied relationship at the level of the dyad members (individuals) and at the level of the dyads highlights the necessity of considering both members of the dyad in assessment. Second, therapists working with infertile couples should be aware of the different dyadic effects; therefore, psychological interventions that target an improvement in marital satisfaction should treat the couple as a unit.

Several limitations of the study should be noted. First, this research was carried out only in one infertility clinic and therefore limits the generalizability of our findings. Second, due to the cross-sectional nature of the study design, casual inference between anxiety and marital satisfaction cannot be made. Further studies, particularly longitudinal research, are required to disentangle the complex associations between spouses’ anxiety and marital satisfaction, including direction of causality and existence of dyadic feedback over time. Other limitations of this study were relatively small sample size, the lack of control for some important clinical variables (i.e., duration of infertility, cause of infertility, and failure of previous treatment), and the lack of specific infertility stress measures.

## Conclusion

In summary, this study highlights the usefulness of DDA to examine and test the phenomena with inherently dyadic nature. We hope that our paper encourages researchers to learn more about dyadic models and how to apply them within the context of behavioral medicine. With regard to our empirical data, the finding confirmed that marital satisfaction in infertile couples was influenced by anxiety at both individual and dyadic level. In addition, the findings showed that a person’s anxiety can impact not only their own but also his/her partner’s marital satisfaction; thus, interventions to improve marital satisfaction should include both men and women. Furthermore, since infertility and its treatments are shared couple problem, future studies should consider using DDA when dyadic data are available in the infertility context.

## Abbreviations

APIM: Actor–partner interdependence model
CFI: Comparative fit index
CFM: Common fate model
DDA: Dyadic data analysis
EMS Scale: ENRICH Marital Satisfaction Scale
HADS: Hospital anxiety and depression scale
MIM: Mutual influence model
RMSEA: Root mean square error of approximation
SEM: Structural equation modeling
SRMR: Standardized root mean square residual
TLI: Tucker-Lewis Index
